# Unicystic Ameloblastoma: A Diagnostic Conundrum

**DOI:** 10.7759/cureus.87870

**Published:** 2025-07-13

**Authors:** Nidhhi Bihani, Sanjay Byakodi, Jyoti M Biradar, Ankita Kadam, Mushtak Khan, Nupur Chawla

**Affiliations:** 1 Oral and Maxillofacial Surgery, Bharati Vidyapeeth Dental College and Hospital, Sangli, IND

**Keywords:** curretage, dentigerous cyst, enucleation, unicystic ameloblastoma, unilocular

## Abstract

Unicystic ameloblastoma (UA), a rare entity among ameloblastomas, is characterized as a tumor that typically occurs in a younger population. This case report aims to emphasize the significance of conducting histopathological examinations on all jawbone lesions, regardless of whether they appear clinically or radiologically insignificant. This study aims to present a case involving UA in a 12-year-old patient, shedding light on its management and follow-up, to assess the prognosis of surgical treatment. The 12-year-old male patient visited the outpatient department. With a complaint of swelling in the right. A maxillary region that had been present for the past three months, which was hard, painless, and measured approximately 6 x 3.5 cm. On Intraoral examination, a painless swelling was observed extending from the right maxillary canine to the distal side of the permanent first molar on the same side. The swelling had a normal texture and consistency, and it was not fixed to the underlying structures. Enucleation with removal of the permanent second molar along with curettage under general anesthesia was the planned treatment, with the differential diagnosis of UA. It was the first diagnosed as a dentigerous cyst and treated with enucleation, removal of the permanent second molar. The actual diagnosis was confirmed after histopathological examination, which revealed Luminal Variant of UA with cystic linings supported by fibrocellular stroma. Cystic lining was composed of a three- to four-layered, four-layer-thick odontogenic epithelium. In these cases, long-term follow-up is necessary as recurrence rates are high. Frequent post-surgical radiographic examinations favor early detection of recurrence.

## Introduction

A wide range of conditions can affect the teeth and the structures involved in their formation. These include developmental anomalies, infections, both bacterial and nonbacterial, injuries, cysts, and tumors. Tumors in the upper jaw, or maxilla, can develop from a variety of non-cancerous growths, either related to tooth development (odontogenic) or unrelated to it (non-odontogenic). Among the odontogenic tumors, ameloblastoma is the most commonly seen. It originates from leftover cells involved in tooth formation and can appear during different stages of dental development. Ameloblastoma is considered a true tumor because it closely mimics the cells that form tooth enamel. Back in 1937, Robinson described it as a benign tumor that usually starts in one area, doesn’t serve any function, grows slowly over time, and while it may not spread, it tends to stick around. Later, in 1991, the World Health Organization (WHO) defined ameloblastoma as a benign but locally aggressive tumor. It tends to come back if not fully removed and is made up of growing tooth-forming cells surrounded by fibrous tissue [1].

In 2017, the WHO updated how we classify certain jaw tumors (specifically, ameloblastomas). They simplified things by grouping them into just three types: conventional, unicystic, and peripheral. To avoid confusion with the unicystic type, the older term solid/multicystic was discarded [2]. Similar radiological and clinical characteristic of an odontogenic cyst is represented in cases of unicystic ameloblastoma (UA). Histological examination reveals a characteristic ameloblastomatous epithelial lining in part of the cystic cavity. Tumor growth may or may not be present within the cyst lining (luminal proliferation) and/or extend into the surrounding wall (mural proliferation). The term *UA* was introduced by Robinson and Martineez in 1977, and it was later referred to as *cystogenic ameloblastoma* by the WHO in the second edition of the *International Histologic Classification of Odontogenic Tumors*. It makes up about 5% to 15% of all ameloblastoma cases, so they are less common compared to the other types [3]. When UA is linked to an unerupted tooth, it typically occurs in patients around 16 years old, whereas in the absence of an unerupted tooth, the average age is 35. There is no noticeable preference for gender in either case [3].

Cystic degeneration of a solid ameloblastoma, development from a dentigerous cyst, or origin from reduced enamel epithelium are different pathological causes of UA [4]. It has been observed in six distinct radiological patterns, ranging from well-defined unilocular lesions to more complex multilocular appearances. However, the unilocular form is most commonly seen, particularly when the lesion is associated with an impacted tooth [5]. Treatment for UA includes options such as radical or conservative surgical removal, curettage, application of chemical agents or electrocautery, radiation therapy, or a combination of surgery and radiation, depending on the specific case and severity [6]. This case report emphasizes the importance of performing histopathological examination for any jaw lesion, even when clinical and radiological evaluations suggest a benign appearance.

## Case presentation

**Figure 1:**
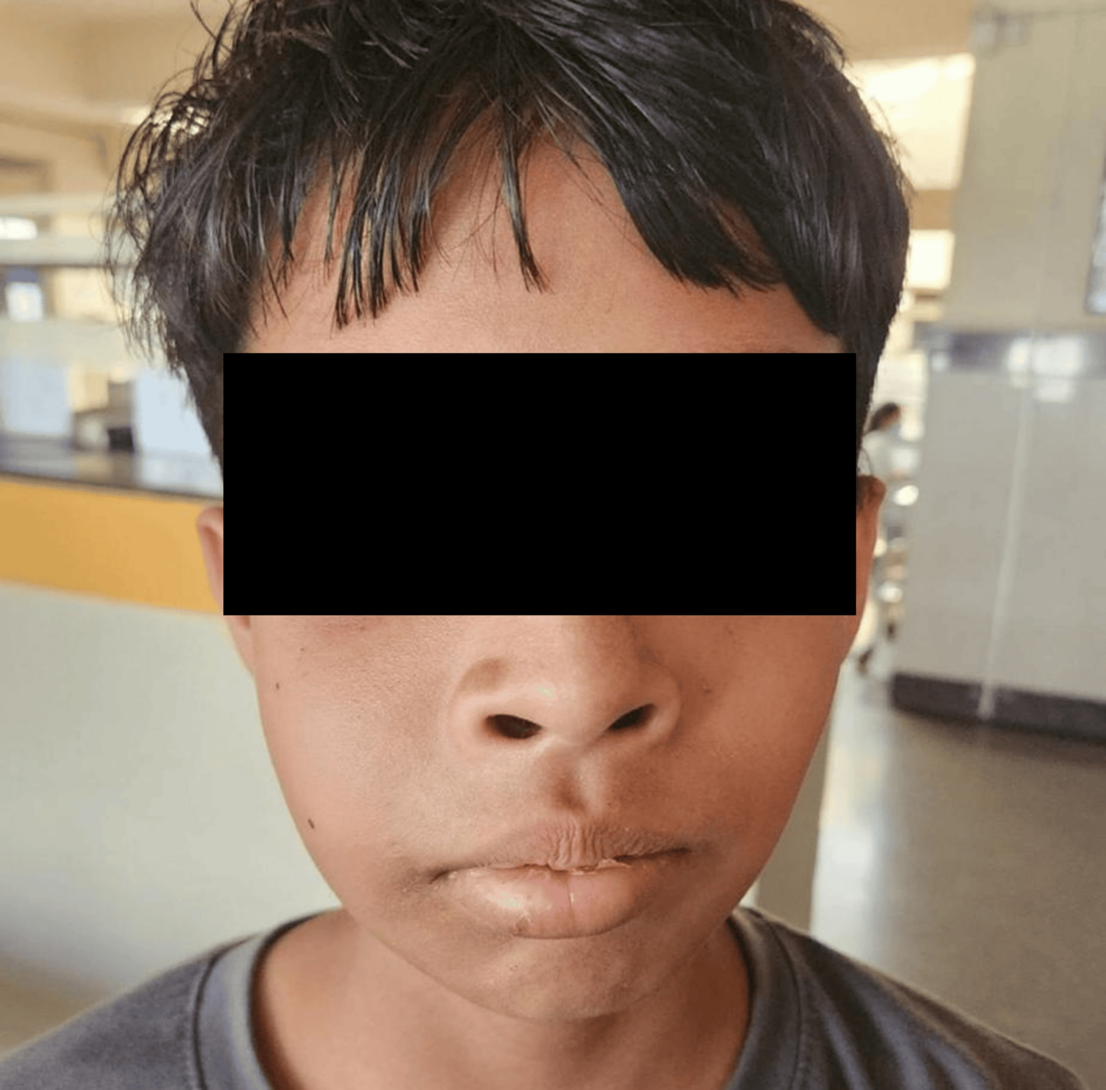
Preoperative profile of the patient: a 12-year-old male showing prominent swelling on the right side of the face.

A 12-year-old male patient visited the outpatient department with the main complaint of a painless, asymptomatic, bony-hard swelling on the right side of his upper jaw, which had gradually increased in size over the last three months. He was alert, cooperative, and fully oriented to time, place, and person, with no significant medical history. Upon clinical examination, a large, non-tender, firm swelling, measuring about 6x3.5 cm, was found in the right maxillary region. The swelling was painless when palpated, and the skin covering it appeared normal in color, texture, and consistency. It was not attached to the underlying tissue (Figure 1).

During the intraoral examination, a painless swelling was noted in the right maxillary region, extending from the canine to the distal part of the first molar on the same side. The swelling was firm to the touch and was covered by mucosa that appeared normal in texture. The second molar was not visible in the oral cavity. Radiographically, the eggshell cracking effect could be appreciated on the buccal aspect of the bone but not over the palatal aspect.

**Figure 2:**
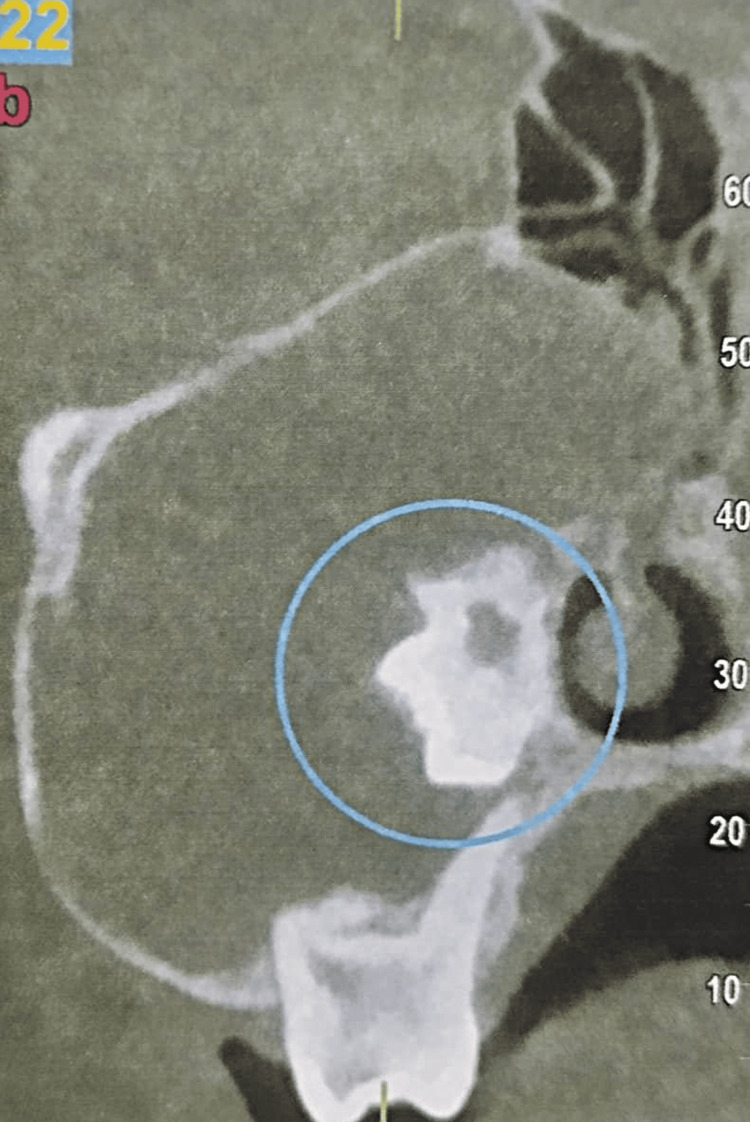
CBCT image showing radiographic evaluation of the ameloblastoma visualized in the scan. CBCT, cone beam computed tomography

Radiographically, a unilocular radiolucent lesion, circumscribed by a radio-opaque border, was observed in the maxillary posterior region, extending from the canine to the maxillary tuberosity. Superior-inferiorly, the lesion extended from the crestal bone to the floor of the orbit, involving the maxillary sinus. The roots of the maxillary right premolars and first molar were displaced from their normal positions, possibly due to cystic pressure. Thinning of the lateral walls of the maxillary sinus was observed, along with impacted maxillary second and third molars displaced within the maxillary sinus. External root resorption was present concerning the maxillary second molar, which was situated toward the lateral wall of the nasal cavity. The lesion involved the sinus ostium and infundibulum, resulting in involvement of the nasal cavity (Figure 2).

The initial diagnosis before surgery suggested that the lesion was likely a dentigerous cyst, based on the patient's age, the location of the swelling, and the presence of an impacted maxillary second molar within the lesion. Enucleation and curettage with removal of the permanent second molar under general anesthesia were planned. The vitality of the teeth involved with the cystic lining was assessed and found to be positive. Root canal treatment was performed on the right maxillary premolars and the maxillary first molar before the surgical procedure.

**Figure 3:**
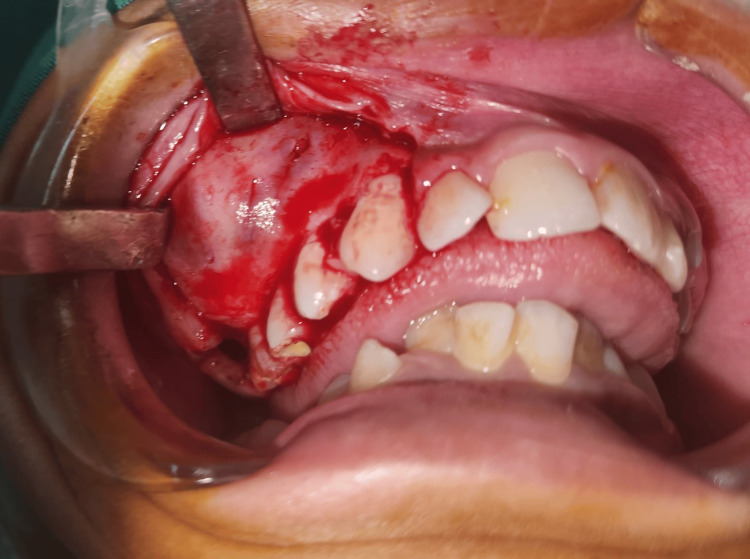
Exposure of the flap: Incision given, followed by exposure of the neoplasm.

An incision was made extending from the distal aspect of the third molar to the medial aspect of the canine. The mucoperiosteal flap was raised, exposing a thin expanded cortical plate, which was removed with the help of a scalpel. The cavity wall comprised mainly of fibrous tissue enclosing liquid within it (Figure 3). The lesion was carefully removed with all its fibrous tissue lining and enucleated to guarantee complete removal. The surrounding bone tissue appeared normal in shape and texture, showing no signs of any other lesions. The maxillary second molar was extracted along with the lesion, and the mucoperiosteal flap was sutured back in place using 3-0 Vicryl after carefully cleaning out the bony cavity with thorough curettage and irrigation.

**Figure 4:**
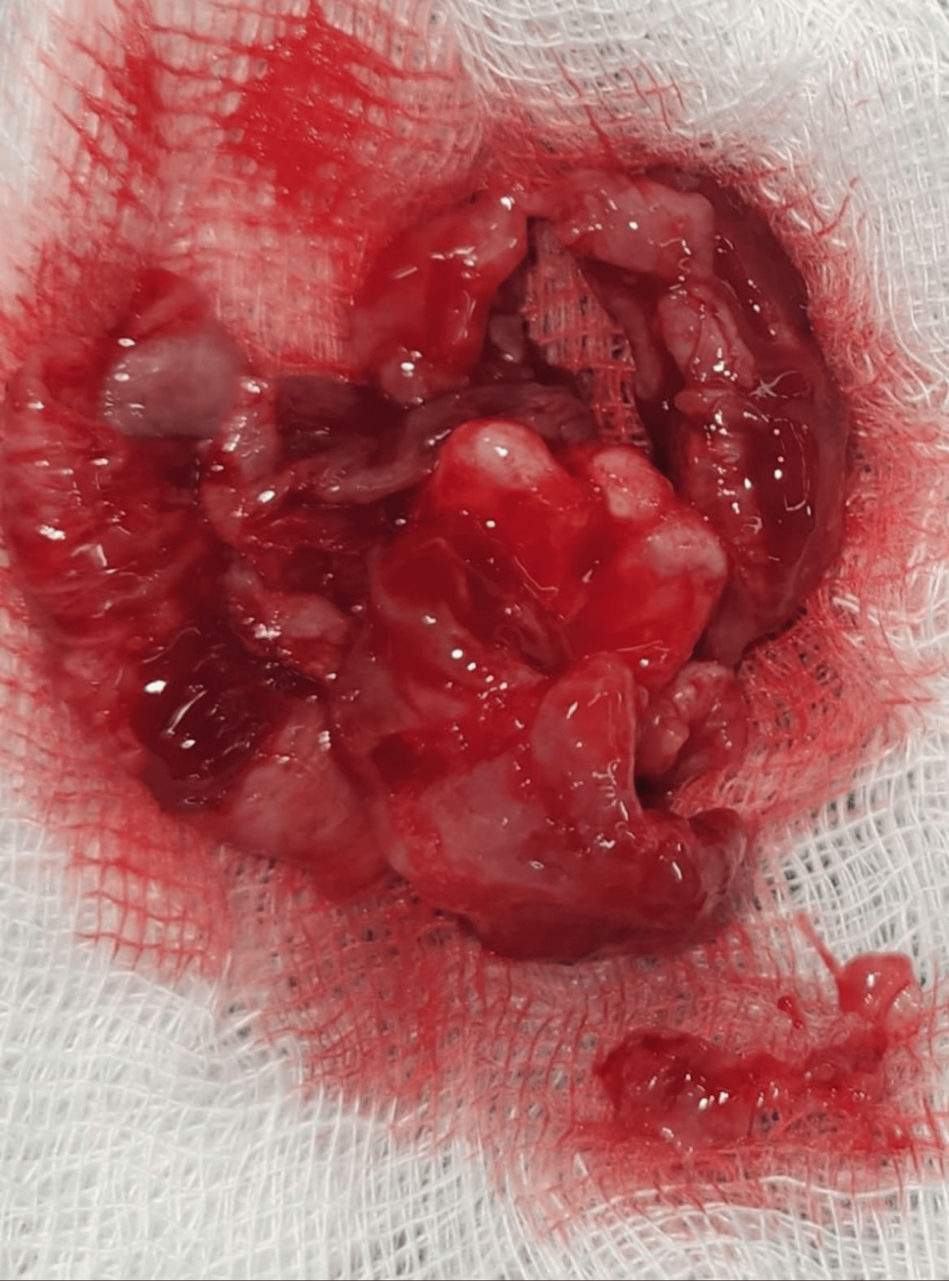
Enucleated cystic content along with the second molar.

The final diagnosis was confirmed through histopathological examination, which revealed a luminal variant of UA, characterized by cystic linings supported by a fibrocellular stroma. The cystic lining consisted of a three- to four-cell-layer thick odontogenic epithelium, which differed from the initial diagnosis of a dentigerous cyst. To monitor any potential recurrence, the patient was placed under radiologic surveillance, as the cyst had been surgically removed with a well-defined cystic envelope, ensuring complete excision (Figure 4).

**Figure 5:**
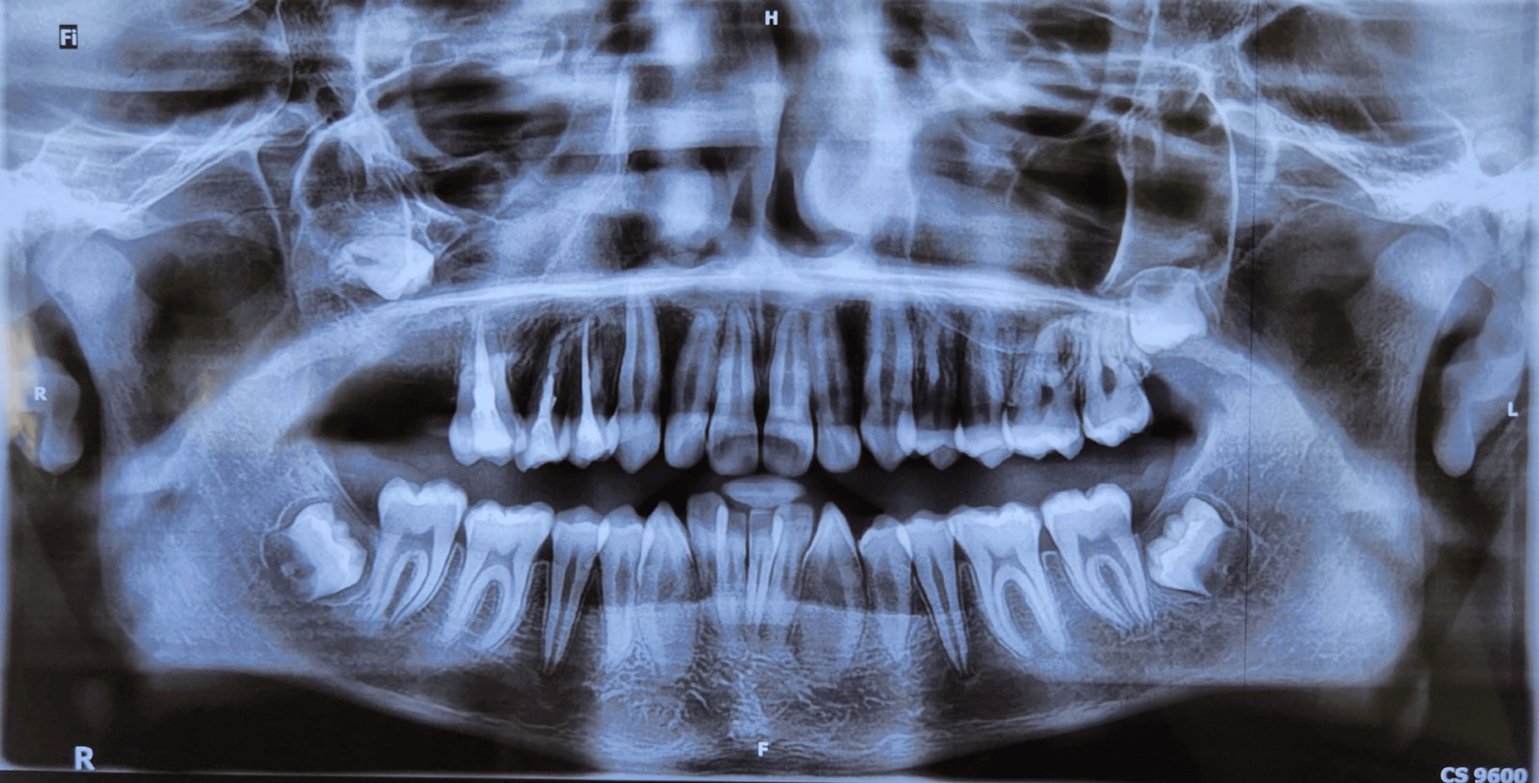
OPG taken at the two-year follow-up to check for recurrence of unicystic ameloblastoma (UA). OPG, orthopantomogram

As of now, the patient has been under follow-up for two years and has shown no signs of recurrence. The affected area has also demonstrated new bone formation. The nature of the cyst was fully explained to the patient, and regular follow-up visits were strongly recommended. No recurrence has been observed during the two-year follow-up period (Figure 5).

## Discussion

Robinson and Martinez first described UA in 1977 as a cystic lesion that exhibits the clinical and radiological characteristics of an odontogenic cyst, but on histological examination shows a typical ameloblastomatous epithelial lining in part of the cystic cavity, with or without luminal and/or mural tumor proliferation [7]. It’s believed to be a rare variant affecting the younger population and is less aggressive, with a favorable response to enucleation and curettage in contrast to solid or multicystic ameloblastomas. Various histological subtypes of UA are recognized, depending on the nature and extent of tumor cell proliferation. These include lesions with a simple cystic architecture and those with intraluminal proliferative nodules, both of which can often be successfully treated with enucleation and curettage. The ones that contain infiltrative tumor islands in the cyst walls should be treated just like solid ameloblastomas, as recurrence following a conservative approach is higher [8].

It is very difficult to differentiate a dentigerous cyst from UA. Some key features that help differentiate UA include painless swelling, unilocular lesions with well-defined sclerotic borders, facial asymmetry, tooth impaction, displacement, mobility, root resorption or divergence, occlusal changes, and extrusion of teeth near the lesion [9]. UA is most commonly found in the mandibular molar and ramus region. Its incidence in the mandible compared to the maxilla is reported at a ratio of 13:1 [10]. In this case, the lesion is associated with the maxillary second molar, presenting as a painless swelling, asymmetry of the face on the right side, impacted maxillary second molar, along with displacement of right maxillary premolars, mobility, or tooth resorption. On radiograph, it is seen as a unilocular radiolucency [11]. All of the characteristics associated with UA were present in our case. Ackermann et al. [11] and Robinson and Martinez [7] suggested that, because the epithelium of odontogenic cysts and ameloblastoma originates from the same source, it is possible, though uncommon, for a non-neoplastic cyst to evolve into a neoplastic one.

Multiple classifications of ameloblastoma are present in the literature. Based on a study of 57 cases of UA, Ackermann et al. classified the tumor into three histologic types:

I. Luminal UA: The tumor is limited to the surface lining inside the cyst.

II. Intraluminal/Plexiform UA: Nodular growths are present within the cyst lumen, but tumor cells do not invade the surrounding connective tissue.

III. Mural UA: Tumor cells invade the connective tissue wall in clusters, but not throughout the entire epithelial lining [11].

Based on this classification, the case in question falls under Group I. There is also another classification by Philipsen and Reichart, which categorizes the different forms of UA as follows [10]:

Subgroup 1: Luminal UA

Subgroup 1.2: Luminal and intraluminal UA

Subgroup 1.2.3: Luminal, intraluminal, and intramural UA

Subgroup 1.3: Luminal and intramural UA

This case report focuses on the surgical treatment of UA in the maxillary region, which has been discussed, as occurrences in this region have seldom been documented in the literature. UA is considered a neoplasm because of its locally aggressive behavior and invasion of surrounding structures, but it lacks the ability of distant metastasize. There are several treatment options for UA, ranging from more extensive procedures like segmental or marginal resection to less invasive approaches. Conservative treatments include enucleation and curettage of the cyst, or initial marsupialization to reduce the size of the lesion, followed by complete removal in a second-stage surgery [7,12,13]. Cryotherapy, thermal or chemical cauterization, radiotherapy, and chemotherapy can be used as adjuncts following the conventional surgical modalities to both promote healing and decrease the chances of recurrence [14].

Determining the best surgical approach to treat this tumor has always been a topic of debate, especially for patients in their second and third decades of life. This adds complexity to treatment decisions right from the initial diagnosis. There is a divide in opinions, with some advocating for more conservative methods like limited resection, enucleation, and curettage, while others argue for more extensive surgical intervention, such as radical resection or maxillectomy [15]. Ameloblastoma in the maxillary region is considered quite rare, as noted in most literature. When patients undergo conservative surgery for treatment, long-term follow-up is essential for monitoring. In cases where UA recurs in the maxillary region, more aggressive treatment, such as radical resection, is often necessary and considered a more suitable surgical approach.

## Conclusions

UA is a rarer form of ameloblastoma and is extremely uncommon in the maxillary region. This case was initially diagnosed as a dentigerous cyst and was treated by enucleation along with removal of the permanent second molar. The actual diagnosis was confirmed after histopathological examination, which revealed a luminal variant of UA with cystic linings supported by fibrocellular stroma. This case report emphasizes the importance of performing histopathological examination for any jaw-related lesion, even if it appears insignificant in both clinical and radiological evaluations. When unilocular radiolucency in the jaw is detected, it's important to closely monitor the condition, conduct thorough examinations, and examine histopathological specimens for a more accurate diagnosis. In these cases, long-term follow-up is necessary as recurrence rates are high. Frequent post-surgical radiographic examinations favor early detection of recurrence.
